# The Dragon’s Paralysing Spell: Evidence of Sodium and Calcium Ion Channel Binding Neurotoxins in Helodermatid and Varanid Lizard Venoms

**DOI:** 10.3390/toxins13080549

**Published:** 2021-08-06

**Authors:** James S. Dobson, Richard J. Harris, Christina N. Zdenek, Tam Huynh, Wayne C. Hodgson, Frank Bosmans, Rudy Fourmy, Aude Violette, Bryan G. Fry

**Affiliations:** 1 Venom Evolution Lab, School of Biological Sciences, University of Queensland, St. Lucia, QLD 4072, Australia; j.dobson@uq.edu.au (J.S.D.); rharris2727@googlemail.com (R.J.H.); christinazdenek@gmail.com (C.N.Z.); 2 Department of Pharmacology, Biomedicine Discovery Institute, Monash University, Clayton, VIC 3800, Australia; tlhuy3@student.monash.edu (T.H.); wayne.hodgson@monash.edu (W.C.H.); 3 Department of Basic and Applied Medical Sciences, Ghent University, 9000 Ghent, Belgium; Frank.Bosmans@ugent.be; 4 Alphabiotoxine Laboratory sprl, Barberie 15, 7911 Montroeul-au-Bois, Belgium; info@alphabiotoxine.be (R.F.); aude.violette@alphabiotoxine.com (A.V.)

**Keywords:** *Heloderma*, *Varanus*, venom, Toxicofera, neurotoxic, sodium channel, calcium channel

## Abstract

Bites from helodermatid lizards can cause pain, paresthesia, paralysis, and tachycardia, as well as other symptoms consistent with neurotoxicity. Furthermore, in vitro studies have shown that *Heloderma horridum* venom inhibits ion flux and blocks the electrical stimulation of skeletal muscles. Helodermatids have long been considered the only venomous lizards, but a large body of robust evidence has demonstrated venom to be a basal trait of Anguimorpha. This clade includes varanid lizards, whose bites have been reported to cause anticoagulation, pain, and occasionally paralysis and tachycardia. Despite the evolutionary novelty of these lizard venoms, their neuromuscular targets have yet to be identified, even for the iconic helodermatid lizards. Therefore, to fill this knowledge gap, the venoms of three *Heloderma* species (*H. exasperatum*, *H. horridum* and *H. suspectum*) and two *Varanus* species (*V. salvadorii* and *V. varius*) were investigated using *Gallus gallus* chick biventer cervicis nerve–muscle preparations and biolayer interferometry assays for binding to mammalian ion channels. Incubation with *Heloderma* venoms caused the reduction in nerve-mediated muscle twitches post initial response of avian skeletal muscle tissue preparation assays suggesting voltage-gated sodium (Na_V_) channel binding. Congruent with the flaccid paralysis inducing blockage of electrical stimulation in the skeletal muscle preparations, the biolayer interferometry tests with *Heloderma suspectum* venom revealed binding to the S3–S4 loop within voltage-sensing domain IV of the skeletal muscle channel subtype, Na_V_1.4. Consistent with tachycardia reported in clinical cases, the venom also bound to voltage-sensing domain IV of the cardiac smooth muscle calcium channel, Ca_V_1.2. While *Varanus varius* venom did not have discernable effects in the avian tissue preparation assay at the concentration tested, in the biointerferometry assay both *V. varius* and *V. salvadorii* bound to voltage-sensing domain IV of both Na_V_1.4 and Ca_V_1.2, similar to *H. suspectum* venom. The ability of varanid venoms to bind to mammalian ion channels but not to the avian tissue preparation suggests prey-selective actions, as did the differential potency within the *Heloderma* venoms for avian versus mammalian pathophysiological targets. This study thus presents the detailed characterization of *Heloderma* venom ion channel neurotoxicity and offers the first evidence of varanid lizard venom neurotoxicity. In addition, the data not only provide information useful to understanding the clinical effects produced by envenomations, but also reveal their utility as physiological probes, and underscore the potential utility of neglected venomous lineages in the drug design and development pipeline.

## 1. Introduction

Voltage-gated ion channels allow the movement of ions across cellular membranes, regulating resting and action potentials. Depending on the type of ions transported, they are categorized as voltage-gated sodium (Na_V_), calcium (Ca_V_), potassium (K_V_), or chloride channels (CLC) [1]. Due to their common evolutionary origin, Na_V_, Ca_V_ and K_V_ channels share architectural similarities [2]. Their α-subunits are comprised of four identical or homologous domains each, with associated voltage-sensing domains (I-IV). Each domain consists of six transmembrane segments (S1–S6). Segments S5–S6 form the ion-selective pore while S1–S4 form the voltage sensor. In Na_V_ channels, voltage-sensing domains I–III are important in channel opening while voltage-sensing domain IV is involved in gating regulation and the fast termination of ion flux post-activation [3,4,5]. Similarly, in Ca_V_ gating, voltage-sensing domains II and III make a major contribution to channel activation, while voltage-sensing domain I are thought to have a minor role in activation [6]. The potential role of voltage-sensing domain IV in Ca_V_ fast inactivation is still debated [6,7].

Ion channels are divided into closely related subcategories with specific physiological roles and are often more highly expressed in particular locations within the nervous system. For example, Na_V_1.4 and Na_V_1.5 are predominantly expressed in skeletal and cardiac muscle, respectively, whereas Na_V_1.6 are found in both the CNS and peripheral nervous system (PNS) [8]. Similarly, the L-type Ca_V_ channels show tissue-selective expression, with Ca_V_1.1 abundant in skeletal muscle and Ca_V_1.2 in cardiac muscle [1,9]. Both Na_V_ and Ca_V_ channels play roles in neurotransmission, endocrine secretion, muscle contraction and sensory perception [10,11,12,13,14]. Therefore, Na_V_ and Ca_V_ channels in physiologically relevant locations have been convergently bound by neurotoxins produced by venomous organisms to aid in prey capture and deter potential predators [15].

Ultimately, ion channel-binding toxins can lead to an increase in, or inhibition of, the release of the cholinergic neurotransmitter acetylcholine. Toxins can bind to the pore region or bind to the various voltage-sensing domains, to inhibit ion channel opening, allow opening at more negative voltages, or delay the inactivation of the channel, thus prolonging ion flux [16]. Common targets of ion channel-binding toxins are the extracellular loops between S3 and S4, a region that flexes in response to membrane depolarizations and drives voltage-sensor activation. As the voltage-sensing domains are structurally similar, toxins that interact with this motif often interact with multiple voltage-sensing domains [17]. Toxins that bind to this locus in voltage-sensing domains I–III typically inhibit channel opening, while toxins that exclusively target voltage-sensing domain IV delay fast inactivation, resulting in spastic paralysis, such as that of the elapid snake *Calliophis bivirgatus* [18]. However, exceptions to this paradigm have been found. A toxin isolated from the tarantula *Hysterocrates gigas* interacts with voltage-sensing domains III and IV to inhibit Ca_V_ channel gating [19]. Similarly, ProTx-II from the tarantula *Thrixopela pruriens* inhibits Na_V_ channel gating via voltage-sensing domains II and IV binding [20], which may cause flaccid paralysis.

Despite their evolutionary novelty, lizard venoms are a neglected area of research.. From a clinical perspective, members of the *Heloderma* genus are an under-investigated venomous reptile lineage partly due to envenomations rarely requiring medical assistance. Bites with serious symptoms are infrequent, often following a bite where the lizard has been attached to the victim for a considerable amount of time, maximizing venom delivery [21,22,23]. This is due to their venom apparatus lacking associated muscles the high-pressure venom delivery seen in snakes. Instead, helodermatid lizards possess mandibular venom glands with multiple ducts leading to the gums at the base of grooved teeth [24]. Therefore, clamping and chewing is required to deliver venom [22].

In addition to coagulotoxic, myotoxic and cytotoxic traits, *Heloderma* bites are often reported to cause pain, paresthesia, tachycardia and paralysis consistent with neurotoxic/cardiotoxic envenomation [23,25,26]. Previous research on *Heloderma* venom has revealed a myriad of protein families often recruited as neurotoxins [24,27,28,29,30]. However, few toxins have been isolated and characterized. Injecting purified helothermine, a Cysteine-rich secretory protein (CRiSP) family toxin isolated from *H. horridum*, produces lethargy, rear limb paralysis and death in rodents [31]. Further studies revealed that helothermine selectively binds ryanodine receptors and blocks K^+^ and Ca^2+^ ion flux in rat cerebellar cells [32,33,34]. Komori et al. (1988) reported on the inhibitory effect of “lethal toxin-1” isolated from *H. horridum* on the direct electrical stimulation of mouse hemidiaphragm [35]. This toxin was later revealed to be constructed of β-defensin domain repeats, thus inducing the renaming of the toxin as helofensin [36]. However, the physiological targets of helofensins and helothermine have yet been determined.

Helodermatid lizards were long thought to be the only venomous lizards. However, studies in many “omics” fields have provided evidence for venom in other anguimorphan lizard lineages, including varanid species, such as the legendary Komodo dragon (*Varanus komodoensis*) [24,37,38,39,40,41]. The majority of varanid lizards are active predators that exhibit extreme differences in size between species: from the 20 cm *V. sparnus* to the over 2 m *V. komodoensis* [42,43]. This disparity in size is also accompanied by variations in diet, hunting behavior and habitat [43,44,45]. Differences in selection pressures due to the variety in the habits of these lizards have led to considerable variation in their venom compositions and activities, rivalling that of the diversity seen within genera of venomous snakes, consistent with active evolution under purifying selection pressure [37,38]. Previous studies incubating varanid venoms with human fibrinogen revealed extensive variation in fibrinogenolytic activity, and other effects include the induction of hypotension [37,38,39,40]. Variable venom activity is reflected in the severity of bites in reports, ranging from minor injuries to hospitalizations requiring medical treatment [46,47,48,49].

The toxins likely responsible for coagulotoxic activities in varanid lizards are kallikrein enzymes, which destructively cleave fibrinogen, and phospholipase A_2_ (PLA_2_), which block platelet aggregation, with both toxin types being homologous to *Heloderma* toxins with the same activity, reflective of their shared molecular evolutionary history and arising from the same venom glands [37,38,40,50,51]. However, many toxin families often recruited as neurotoxins in other organisms, such as AVIT, PLA_2_, cysteine rich secretory proteins (CRiSPs) and kunitz peptides, have been identified in the venom gland transcriptomes or venom proteomes of varanid and helodermatid lizards [15,24,27,28,29,30,37,40]. Indeed, the neurotoxic peptides in the AVIT and cholecystoxin classes were likely responsible for the strong contraction of rat smooth muscle after incubation with *V. varius* venom [37,38]. In addition, a study noted that injecting birds and rodents with crude *V. griseus* venom resulted in immediate paralysis of rodents, suggesting the presence of taxon-selective receptor-binding neurotoxins [48,52]. 

Human envenomations by varanid lizards that present with neurotoxic symptoms are rare, but have been reported. Due to the similarities of their venom apparatus to those of *Heloderma,* symptoms of envenomation by varanids follow incidents where the lizard has been attached to the victim for considerable time [46,47,48]. Whilst bites from larger species such as *V. varius* and *V. salvadorii* have anticoagulant effects, any neurotoxic symptoms caused may be masked by the pain caused by the often severe mechanical damage these species can inflict [49]. However, bites from *V. griseus* have been reported to produce specific symptoms (muscle weakness, local and systemic pain, respiratory distress and difficulty walking), and tests of the venoms on animal models have confirmed these actions [46,47,48]. The site of action, however, remains enigmatic. Similarly, other victims have observed neurotoxic symptoms post-bites from *V. scalaris*, comparing the sensation to that of a paper wasp sting [44,49]. 

Despite the evidence presented for neurotoxins within varanid lizard venoms, few studies have investigated the activity of the venom on assays assessing neurotoxicity. Furthermore, studies have yet identified the neurological targets of *Heloderma* venom neurotoxins. Therefore, to fill these knowledge gaps, we investigated the neuropathology of helodermatid and varanid lizard venoms. To observe the effects on whole receptors, we used the *Gallus gallus* chick biventer nerve–muscle preparation assay, an avian skeletal muscle preparation well-validated through numerous studies on snake venoms [18,53,54,55,56]. To determine the binding affinity of the venoms for various ion channels, we used biolayer interferometry to ascertain binding to the voltage-sensing domain IV S3–S4 extracellular loop mimotopes for human sodium and calcium channels, an approach validated using venoms as diverse as snakes and stonefish [57,58,59,60,61,62,63]. The present study represents the most rigorous investigation of the ion channel neurotoxicity of the *Heloderma* genus, and the first for varanid lizard venoms. Information regarding the fundamental biochemical actions of the venoms may be of use in the evidence-based design of clinical management strategies for envenomed patients. In addition, the results may reveal useful probes of physiological pathways, or reveal novel lead compounds for the drug design and development pipeline. 

## 2. Results

### 2.1. Gallus gallus Chick Biventer Cervicis Nerve–Muscle Assays

**Figure 1:**
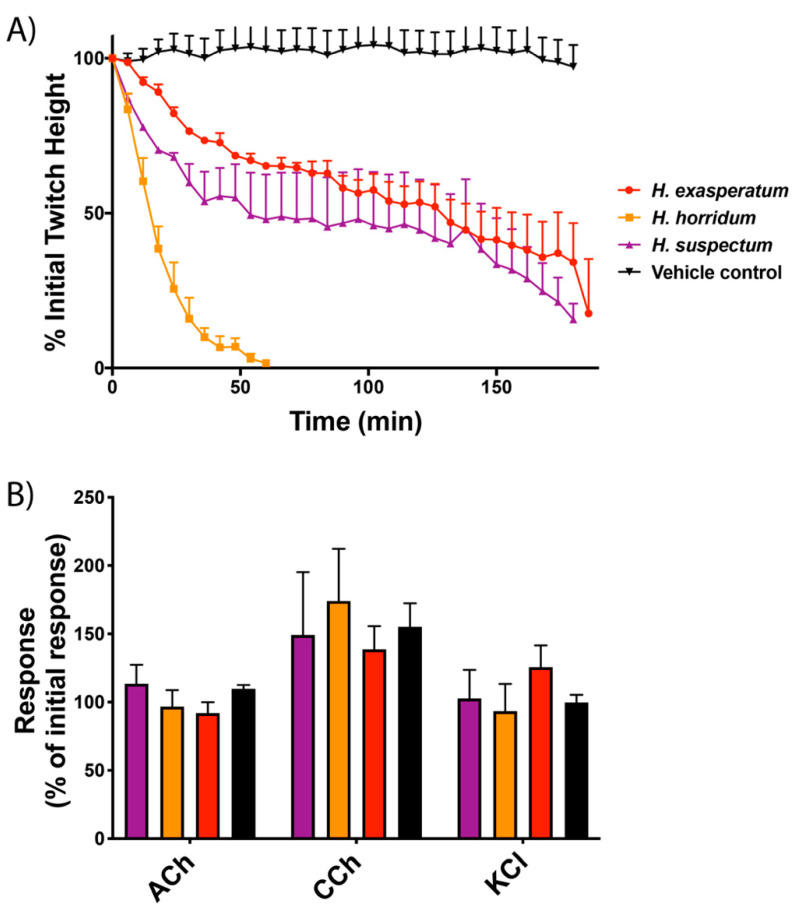
*Gallus gallus* chick biventer cervicis nerve–muscle preparation results after incubation with *Heloderma* venoms; (**A**) inhibition of indirect muscle twitches by *Heloderma exasperatum* (red), *H. horridum* (orange) and *H. suspectum* (purple) venoms relative to the initial twitches recorded prior to venom addition. Vehicle control shown in black; (**B**) effect of venoms on the contractile responses of the agonists acetylcholine (ACh), carbachol (CCh) and potassium chloride (KCl) relative to the initial response. All experiments were performed in triplicate.

Testing on the avian neuromuscular preparation revealed that the muscle twitch response heights were markedly attenuated when *Heloderma* venoms were added (Figure 1). The most potent of the three *Heloderma* venoms was *H. horridum*, which abolished twitches within 60 min (Figure 1A). The venoms of *H. exasperatum* and *H. suspectum* had similar activities, inhibiting twitches by approximately 80% over the time-course of the experiment (Figure 1). There were no significant differences between responses to the agonists acetylcholine (ACh), carbachol (CCh) and potassium chloride (KCl) in the presence or absence of the venoms, with all groups producing responses comparable to the initial contractile response (Figure 1B). Parallel experiments run with *V. varius* venom did not produce notable results at the concentrations tested on this avian tissue preparation (see Supplementary Data).

### 2.2. Biolayer Interferometry Assays

**Figure 2:**
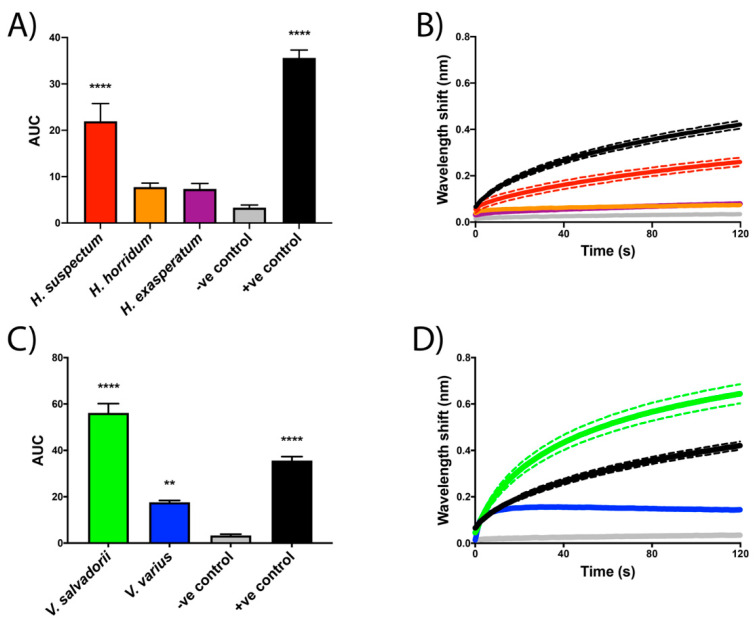
Binding affinity of *Heloderma* (**A**,**B**) and *Varanus* (**C**,**D**) crude venoms to the S3–S4 extracellular loop mimotope of domain IV of the human sodium channel Na_V_1.4. Figures (**A**,**C**) depict the area under the curve (AUC) of the curves displayed in figures (**B**,**D**). The Y axis of figure (**B**) shows the wavelength (nm) from the association (Ka binding) step. Asterisk symbols (*) above the bars (**A**,**C**) denote the level of statistical significance relative to the negative control. The negative control (-ve) was *Naja kaouthia* crude venom, while the positive control was *Leiurus quinquestriatus* crude venom. Dotted lines surrounding curves represent the error bars (**B**,**D**) based on SEM values from experiments performed in triplicate.

**Figure 3:**
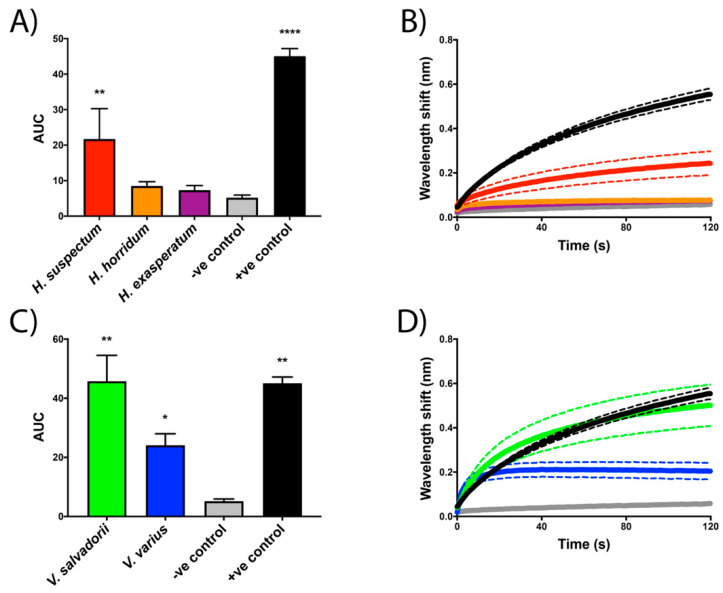
Binding affinity of *Heloderma* (**A**,**B**) and *Varanus* (**C**,**D**) crude venoms to the S3–S4 extracellular loop mimotope of domain IV of the human calcium channel Ca_V_1.2. Figures (**A**,**C**) depict the area under the curve (AUC) of the curves displayed in (**B**) and (**D**). The Y axis of (**B**) shows the wavelength (nm) from the association (Ka binding) step. Asterisk symbols (*) above the bars (**B**,**D**) denote the level of statistical significance relative to the negative control. The negative control (-ve) was *Naja kaouthia* crude venom while the positive control was *Leiurus quinquestriatus* crude venom. Dotted lines surrounding curves (**C**,**D**) represent the error bars based on SEM values from experiments performed in triplicate.

As it is a well-described ion channel-attacking venom, *L. quinquestriatus* was used as a positive control to validate the assay parameters, displaying strong binding to the S3–S4 extracellular loop mimotope of voltage-sensing domain IV of human Na_V_1.4 and Ca_V_1.2 (Figure 2A,B and Figure 3A,B, respectively). Wavelength response shifts indicate *Heloderma suspectum* venom binds strongly to the voltage-sensing domain IV motif of human Na_V_1.4 (Figure 3A,B) and Ca_V_1.2 (Figure 3A,B). In contrast, the other helodermatid lizard species tested, *H. exasperatum* and *H. horridum,* did not display significant binding to these mammalian targets (Figure 2 and Figure 3). However, wavelength response shifts indicate that *V. salvadorii* venom strongly binds to the voltage-sensing domain IV motif of both Na_V_1.4 (Figure 2C,D) and Ca_V_1.2 (Figure 3C,D). Although still binding significantly, *V. varius* venom was less potent than *V. salvadorii* on both targets, and proportionally weaker on Ca_V_1.2 than Na_V_1.4 (Figure 2C,D), being equipotent to *H. suspectum* on Ca_V_1.2 (Figure 3) but slightly less potent than *H. suspectum* on Na_V_1.4 (Figure 2).

## 3. Discussion

Helodermatid lizard venoms have previously been shown to inhibit the electrical stimulation of mouse hemidiaphragm and induce paralysis in mice [31,35], but the site of action was not determined in this prior work. Here, we present similar results displaying a reduction in contraction ability in avian skeletal muscle (Figure 1A). Responses to postsynaptic agonists suggest *Heloderma* venoms act pre-synaptically, with the absence of myotoxicity (Figure 1B). Congruent with the avian tissue preparation results of presynaptic neurotoxicity, biolayer interferometry assays revealed *Heloderma suspectum* bound to the S3–S4 loop of voltage-sensing domain IV within Na_V_1.4, inhibiting channel fast inactivation and altering Na^+^ influx (Figure 2A,B). While many toxins that bind to voltage-sensing domain IV have a stimulatory effect, such as delaying inactivation [18], binding to produce an inhibitory effect has been documented [20], thus supporting our results. In addition, many toxins found to bind to voltage-sensing domain IV also interact with voltage sensing domains I-III, where binding would inhibit gating [17]. The dichotomy between the avian tissue preparation and the mammalian-target biolayer interferometry assays is suggestive of differential taxon-specific effects between the three *Heloderma* species tested and thus is a rich area for future research regarding the evolutionary selection pressures leading to such lineage-selective effects.

While *V. varius* venom did not produce discernable results on the avian-tissue preparation assays at the concentrations tested, both *V. varius* and *V. salvadorii* bound mammalian Na_V_1.4 with *V. varius* nearly equipotent to *H. suspectum* while *V. salvadorii* was almost twice as potent (Figure 2). These results support the clinical symptoms of lethargy and paralysis reported in the bites from helodermatid and, occasionally, varanid lizards [21,46,48]. Further support can be found from previously conducted in vivo studies [31,35,52]. *Varanus griseus* venom is reported to produce paralyzing effects on mice, consistent with Na_V_1.4 antagonistic activity, similar to the effects of helothermine [31,52]. The study by Gorelov (1971) also injected sparrows with *V. griseus* venom, which did not result in the immediate paralysis observed in mice, corroborating the lack of effect of the varanid venoms on the avian tissue preparation assays and the strong binding in mammalian target assays.

The disparity between avian and mammalian data is suggestive of differences in ion channel physiology between organisms. Structural differences between voltage-gated ion channels of avian and mammalian species (as shown in the sequence variation of key domains in uniprot accessions P35499 and P35499) could explain the lack of venom activity displayed by the varanid lizards in avian tissue preparation assays and the strong binding in mammalian biolayer interferometry assays, and the converse where *H. exasperatum* and *H. horrium* were much more potent in the avian assay than the mammalian (Figure 1, Figure 2 and Figure 3). As the *Heloderma* and *Varanus* species studied here are known to consume both mammals and birds, different toxin classes may be selected for the subjugation of different prey, as has been observed for snake venoms [64]. Such differences observed for snake venoms include extreme variations between prey lineages, suggestive of taxon-specific effects [65,66,67,68,69,70,71]. Other lineage-specific effects include the binding affinity of μ-conotoxins, which are significantly more potent binders of frog TTX-resistant Na_V_ channels as compared to homologous channels in rat and mouse assays and while such variation is not reflective of any sort of biological reality (as cone snails do not feed on frogs or rodents) this specificity of venoms never-the-less underscores the potential use of venoms as probes for differences in physiological pathways of disparate animal lineages [72,73]. 

Compared to other *Heloderma* venoms tested, *H. horridum* venom was more potent on avian tissue preparation assays (Figure 1) and showed a lower binding affinity to the mammalian ion channel domains, possibly reflecting a predatory role of the venom with variations in diet between *Heloderma* species. Due to the elusive subterranean nature of *Heloderma* species, studies on diet comparisons are lacking, often limited to seasonal above-ground observations. Thus, the abundant observations of helodermatid lizard diets preying upon defenseless prey, such as eggs and nestlings [25,43,74], are likely due to the opportunistic nature of these observations when the lizards are feeding upon birds and eggs during annual avian breeding periods. Supporting this supposition are records of *H. suspectum* consuming mammals, which rapidly succumb to the venom’s effects ([75], Fry’s personal observations).

An alternative testable hypothesis is that differences in venom activity between species could be selected for defensive purposes. *Heloderma suspectum* are the smaller of the species tested and thus are likely more vulnerable to mammalian predators, such as *Canis latrans* [25]. This is consistent with the more prominent aposematic markings found on *H. suspectum*, indicating that a bite causes pain. Indeed, severe pain is often recorded in bite reports from this species [76]. Many varanid lizard species are also vulnerable to mammalian and avian predators as juveniles, and for smaller species, as adults; therefore, a painful bite would confer an advantage [43]. It is unclear if the activity presented in this study may contribute to the pain from a bite, as there is extensive cross-reactivity between sodium channel types, and thus paralytic toxins binding to the neuromuscular target Na_V_1.4 may also bind to other sodium channels involved in pain. However, the interactions of Na_V_1.4 shown in this study would produce paralysis of skeletal muscles, such as is shown in the tissue preparation, which is consistent with evolutionary selection pressures for a predatory role. Thus, a testable hypothesis for future work is that *Heloderma* venom contains components for prey subjugation, in addition to serving a defensive role due to the cross-reactivity between sodium channel subtypes. 

Commonly reported in severe *Heloderma* human bite clinical cases are tachycardia, arrythmia, and the potentially fatal cardiac ischemia [22,23,76]. An increased, irregular heart rate has also been observed following varanid lizard bites [46,48]. As Ca_V_1.2 is primarily a cardiac muscle ion channel, binding to this channel could be the mode of action responsible for these symptoms. Harris et al. (2021) reported on the binding of stone fish (*Synanceia verrucosa*) venom to the S3–S4 extracellular loop of voltage-sensing domain IV of Ca_V_1.2, suggesting this activity could explain the arrythmia and tachycardia that manifest post-stone fish envenomation. In addition, severe scorpion envenomations can cause cardiac ischemia [77]. A direct cardiac effect may act synergistically with the mass release of catecholamines that has been suggested as the cause of similar symptoms seen in *Heloderma* bite victims [76]. Thus, the role of the Ca_V_1.2 binding effect is another rich area for future research into the predatory or defensive ecology of anguimorphan lizards. In addition to the calcium channel-binding toxins, the effects upon heart rate may also be due to the sodium channel-binding toxins binding to the cardiac-associated Na_V_1.5 channel [8]. While not investigated in this study, toxins that bind to Na_V_1.4 have also been reported to bind to other Na_V_ channels, including Na_V_1.5 [78]. Thus, the ion channel interactions responsible for cardiac dysfunction is another rich area of future research.

Venom binding to both Na_V_ and Ca_V_ channels is often due to the possession of a complex cocktails of toxins which individually may have selective binding affinities for either channel family. Conversely, some toxins are promiscuous in being able to bind across not only Na_V_ or Ca_V_ channel subtypes, but due to the homologous architecture from a shared molecular evolutionary history, some ion channel toxins are able to act upon both Na_V_ and Ca_V_ channels. Examples include hanatoxin, which binds to multiple subtypes of Na_V_, Ca_V_ and K_V_ channels [16]. Similarly, other tarantula venom peptides have been found to be active on both Na_V_ and Ca_V_ channels [79]. Thus, the structure-function responsible for such diverse binding by some venom toxins is a rich area for future research.

Based on the results presented in this study, future work should investigate other species of varanid lizards for their potential neurotoxic venom activities. While we were limited by species available, experiments should be performed on species previously shown to produce neurotoxic activities and symptoms, such as *V. griseus*, *V. scalaris*, and other closely related species. Binding to the S3–S4 extracellular loop of domains I-III could potentially be investigated using biolayer interferometry mimotope assays similar to those used in this study. Despite evidence of toxins binding to the voltage-sensing domain IV of Na_V_1.4 and Ca_V_1.2, translating these data into the effects on whole ion channel gating would require future work on additional testing platforms, such as the K_v_ channel chimeric approach or electrophysiological techniques such as oocyte patch-clamp assays. In addition, future work should include fractionating the venoms to ascertain the toxin types responsible for these intriguing activities. 

In summary, we have elucidated the calcium and sodium channel-binding mechanisms of *Heloderma* venom, and report for the first time evidence of ion channel binding neurotoxins in *Varanus* venoms. This work provides a foundation for natural history investigations to ascertain the biological roles of lizard venoms, whether as predatory weapons or as part of a defensive arsenal, or a combination thereof. Notably, the neurotoxic effects revealed in this study are indicative of both defensive and predatory roles for the venoms of *Heloderma* and *Varanus* species, and further suggest prey-selective effects of both *Heloderma* and *Varanus* venoms. The modes of action of these venoms presented in this study could also be important in understanding the clinical effects of envenomations, by revealing the underlying biochemistry of neurotoxic symptoms, thereby providing data which may be useful for the evidence-based design of clinical management strategies. The results further underscore the utility of using venoms as rich sources of novel compounds, useful as probes to ascertain physiological function, and even as lead compounds for drug design and development. Animal toxins have been used as key probes in the discovery of voltage-gated ion channel subtypes, their structural properties, and their functional characteristics. Our results reinforce the use of biolayer interferometry assays as a potential tool to investigate the role of voltage-sensing domains in ion channel gating. Thus, in addition to being selective probes for ion channel functions, these novel compounds may have utility as starting substrates in the drug design and development pipeline. It is only by investigating the full range of venomous animals, such as the neglected venomous lizard lineages, that such resources can be harnessed to their fullest potential.

## 4. Materials and Methods

### 4.1. Sample Acquisition

Venoms were collected by encouraging the specimens to chew on soft rubber tubing, with the mandibular secretions collected with pipettes (*Varanus*) or allowed to flow into a 50 mL tube (*Heloderma*). Secretions were then centrifuged at 14,000 RCF (4 °C, 10 min.) to remove insoluble material, filtered with a 40 µm syringe filter, flash-frozen in liquid nitrogen, and then freeze-dried. *Varanus varius* samples (adult male, *n* = 3, pooled) were collected from captive specimens under University of Queensland animal ethics approval SBS/403/16 (approval date: 1 February 2016). Non-Australian lizard (*H. exasperatum*, *H. horridum*, *H. suspectum*, and *V. salvadorii*) samples were supplied by licensed biotechnology company Alphabiotoxine Laboratory, Montroel-au-bois, Belgium.

### 4.2. Gallus Gallus Chick Biventer Cervicis Nerve–Muscle Assays

Neurotoxicity was ascertained using a previously validated organ bath protocol [80]. *Gallus gallus* chicks aged between 4–10 days were euthanized with CO_2_ (animal ethics number 22575 was approved by Monash University Ethics Committee on 18 December 2019). Biventer cervicis nerve–muscle samples were removed and mounted under 1 g tension in 5 mL organ baths containing physiological salt solution (NaCl, 118.4 mM; KCl, 4.7 mM; MgSO_4_, 1.2 mM; KH_2_PO_4_, 1.2 mM; CaCl_2_, 2.5 mM; NaHCO_3_, 25 mM; and glucose, 11.1 mM). Organ baths were bubbled with carbogen (95% O_2_; 5% CO_2_) at a constant temperature of 34 °C. Electrodes were placed around the tendon of the biventer muscle and electrical stimulation at the motor nerve (0.2 ms duration, 0.1 Hz, supramaximal V) using a Grass S88 stimulator (Grass Instruments, Quincy, MA, USA) evoked indirect twitches. Selective stimulation of the nerve was confirmed by the abolition of twitches with d-tubocurarine (10 µM), a nAChR competitive antagonist. Tissues were then washed repeatedly with physiological salt solution to restore twitch responses to nerve stimulation. The stimulation was ceased, and the contractile responses to acetylcholine (ACh, 1 mM for 30 s), carbochol (CCh, 20 µM for 60 s), and potassium chloride (KCl, 40 mM for 30 s) were obtained and recorded. The organ bath was then washed, and electrical stimulation was resumed and maintained for 30 min to allow the preparation to equilibrate. Venom (10 µg/mL) was added to the organ bath, and the twitch height was recorded until the abolition of twitch response or was stopped after a 1 h period. The stimulator was turned off again and the bath was washed. Contractile responses to ACh, CCh and KCl were obtained again to compare with responses prior to venom addition. The twitch responses to electrical stimulation and contractile responses to agonists (ACh, CCh, and KCI) were measured using a Grass FT03 force displacement transducer (Grass Instruments, Quincy, MA, USA) and recorded on a PowerLab system (ADInstruments Pty Ltd., Bella Vista, NSW, Australia). The data were then converted into figures using Prism8.0 software (GraphPad Software Inc., La Jolla, CA, USA).

### 4.3. Biolayer Interferometry

**Figure 4:**
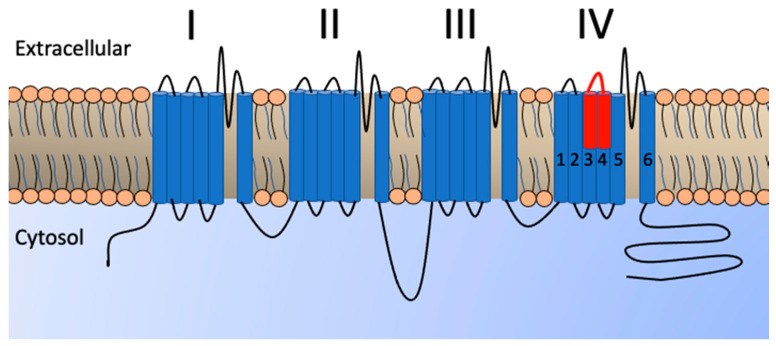
Graphic depicting the basic structure of a voltage-gated ion channel α-subunit displaying the position of the voltage-sensing domains I–IV in the phospholipid bilayer. Blue cylinders represent transmembrane helices while black lines represent connective loops. Highlighted in red is the S3–S4 extracellular loop of voltage-sensing domain IV used in the biolayer interferometry assays. As previously validated [57,80], biosensor mimotopes were designed from sequences for this region, as follows: for Ca_V_1.2 (Homo sapiens), AEHTQSSPSMNAEENSRISITFFRLFRVMRLVK, and for Na_V_1.4 (Homo sapiens), SIVGLALSDLIQKYFVSPTLFRVIRLARIGRVLR.

The voltage-sensing domain IV S3–S4 motifs were ascertained using previously validated protocols [57,81]. The amino acid sequences for human voltage-sensing domain IV S3–S4 motifs are shown in Figure 4 for Ca_V_1.2 (Homo sapiens, uniprot accession code Q13936) and Na_V_1.4 (Homo sapiens, uniprot accession code P35499). These regions were synthesized by GenicBio Ltd. (Shanghai, China) and joined to two aminohaxanoic acid (Ahx) spacers forming a 30 Å linker, with the end Ahx then bound to biotin, providing clearance between the biotin and mimotope and thereby allowing the mimotope to maintain its natural conformational freedom when binding to the analyte in solution. Dried stocks of mimotopes were solubilized in 100% dimethyl sulfoxide (DMSO) prior to dilution with deionized water in a 1:10 ratio to produce a working stock concentration of 50 µg/mL. This stock was then stored at −80 °C until required for experiments. Binding kinetics were analyzed by biolayer interferometry utilizing the Octet HTX system (ForteBio). All assays were conducted in standard Greiner black 96-microtiter well plates (ref: 655209). Analyte (venom) experimental concentrations of 50 µg/mL (10 µg per well) were produced by diluting stock solutions 1:20 with matrix buffer (1× DPBS with 0.1% BSA and 0.05% Tween-20). Mimotope aliquots were diluted 1:50 with matrix buffer to produce a final concentration of 1 µg/mL (0.2 µg per well). Streptavidin sensors were hydrated in the matrix buffer for 30–60 min, whilst being agitated at 2.0 RPM on a shaker prior to experiments. A standard acidic solution glycine buffer (10 mM glycine (pH 1.5–1.7)) in deionized water was used to regenerate the sensor tips during experimentation while still leaving the mimotope attached to the sensor. Controls consisted of a buffer control (deionized water:glycerol 1:1) and the negative control *Naja kaouthia* (monocled cobra) venom in the place of the analyte sample in the wells. The positive control for Na_V_1.4- and Ca_V_1.2-binding was *Leiurus quinquestriatus* (deathstalker scorpion) venom, which is known to strongly bind to both channels. 

Data were processed in accordance with the validation study [59]. The association step data (.csv file) were extracted for each triplicate and imported into Prism 9.0 software (GraphPad Software Inc., La Jolla, CA, USA) where area under the curve (AUC) calculations were made and data graphed. Dunnett’s multiple comparison tests were performed to determine statistical significances between treatment groups and negative controls.

## Data Availability

The data presented in this study are available in supplementary material.

## Supplementary Materials

The following are available online at https://www.mdpi.com/article/10.3390/toxins13080549/s1, S1: Raw Data.
